# Quadriceps vascular occlusion does not alter muscle action or balance: A cross-sectional study

**DOI:** 10.4102/sajp.v80i1.1954

**Published:** 2024-01-31

**Authors:** Daiene C. Ferreira, Letícia B. Vale, Felipe H. Santos, Christiane S.G. Macedo

**Affiliations:** 1 Department of Physiotherapy, State University of Londrina, Londrina, Brazil

**Keywords:** electromyography, vascular occlusion, quadriceps, muscle strength, postural balance, women

## Abstract

**Background:**

Partial vascular occlusion (PVO) can increase muscle strength and hypertrophy without joint overload. However, PVO could increase the possibility of imbalances and injuries during physical activity.

**Objectives:**

To identify changes in strength, muscle activation, and postural control during the use of PVO in young women.

**Method:**

A total of 14 healthy women aged between 18 and 30 years were evaluated. Dynamometry was used to analyse the strength of the quadriceps muscle, and surface electromyography to evaluate quadriceps muscle activity. A force platform was utilised to assess postural control, static single-legged support, single-legged squat, and climbing and descending stairs. Participants were randomly assigned to the evaluations either with or without PVO. The results were compared and correlated.

**Results:**

The performance of static, dynamic, or stair exercises, with or without PVO, did not indicate differences in muscle strength and recruitment (*p* > 0.05). The use of PVO improved the velocity of anteroposterior (AP) oscillation of static postural control (*p* = 0.001). We found a moderate negative correlation between muscle strength and postural control during the ascending stairs task with the use of PVO (*r* = −0.54; *r* = −0.59), while in the group without PVO, the correlation was moderate to high (*r* = −0.55; *r* = −0.76).

**Conclusion:**

The use of PVO did not impair muscle strength and recruitment of the quadriceps or postural control in healthy women.

**Clinical Implications:**

Partial vascular occlusion can be used during dynamic exercises without impairing the balance and muscle strength of the quadriceps during its execution.

## Introduction

Muscle strength and endurance training contribute to the prevention of musculoskeletal injuries and dysfunctions, as well as delaying the onset of age-related diseases (Hughes, Ellefsen & Baar 2018). The American College of Sports Medicine (ACSM) recommends loads of 60% – 70% of one maximum repetition (1RM) for strength gain, and 70% – 85% of 1RM for hypertrophy (Miller et al. 2021; Ratamess et al. 2009; Wortman et al. 2021). However, exercises with high loads can lead to stress and injuries to muscles, tendons, and joints (Forte et al. 2021).

The use of partial vascular occlusion (PVO) has been proposed to increase muscle strength and hypertrophy, and avoid overloading the musculoskeletal system (Álvarez et al. 2021). The practice of exercises with PVO decreases the time to exhaustion, explained through the processes of muscle fatigue that occur early because of the lack of oxygen and failure in oxidative capacity, which causes an accelerated decline in muscle fibre strength (Willberg, Zentgraf & Behringer 2021). Humes et al. (2020) confirm that an intramuscular hypoxic environment can induce vascular endothelial growth and high levels of metabolic stress, which may lead to hypertrophy. Additionally, increases are observed in the concentration of growth factors, satellite cells, transcription factors, reactive oxygen species, intramuscular anabolic signalling, anticatabolic reactions, and recruitment of type II muscle fibres, which could facilitate muscle hypertrophy, with results similar to those of classic muscle mass gain, but with reduced joint stress and, therefore, increased exercise tolerance (Nakajima et al. 2007; Takarada et al. 2000). Hence, PVO enables the achievement of the same results with lower loads (approximately 20% – 30% of 1RM), avoiding joint overload, and preventing injuries and/or dysfunctions in the musculoskeletal system (Clark et al. 2011; Ellefsen et al. 2015).

Partial vascular occlusion or partial blood flow restriction is a method that uses a cuff and/or tourniquet placed on the proximal area of the limb, and then, when inflated, partially restricts the blood flow to the muscles involved in the movement (Cognetti, Sheean & Owens 2022). It is reported that there are hypoalgesia responses after low load resistance exercise with PVO (Korakakis, Whiteley & Epameinontidis 2018), with effects maintained for up to 24 h, which highlights the result of exercise analgesia associated with PVO (Hughes, Stephen & Patterson 2020). In addition, there are positive repercussions in improving muscle strength and endurance of young and older individuals (Centner et al. 2019; Lixandrão et al. 2018; Slysz, Stultz & Burr 2016), with no difference in gain compared with high load training (Early et al. 2020) and the advantage of less pain and discomfort when performing movements, especially for individuals in rehabilitation.

Although the positive effects in the medium or long term after starting training with PVO are well established, little is known about the immediate effects or the effects during the execution of the exercises because the practice of PVO decreases the blood flow and, consequently, the supply of oxygen to the active muscle. It is not yet known if the performance of exercises with PVO presents any risk or deficits, whether muscle activation is differentiated, or if postural control is changed. It is therefore necessary to investigate the effects during the execution of these exercises on muscle strength gain and recruitment, as well as postural control in healthy individuals. We hypothesised that, in the presence of changes in postural control, muscle strength, or activation in the execution of exercises with PVO, there could be greater postural imbalance and less safety during the performance of the exercise, with an increased probability of overload or injury. To address this gap in the literature, our study analyses whether the PVO of the proximal thigh alters the strength and muscle recruitment of the quadriceps, as well as the postural control of young women.

## Methods

Our cross-sectional study included healthy women, and non-athletes, aged between 18 and 30 years, with no musculoskeletal complaints. Exclusion criteria, namely, previous surgical procedures, changes in plantar sensitivity, vascular diseases, and coronavirus disease 2019 (COVID-19) were established in the 6 months before the evaluation.

The calculation of the sample size was based on the study by Centner et al. (2019) and considered the score of muscle strength in the *leg press* exercise, with values of 1180.1 ± 250.1 *n* in the pre-intervention group and 1190.8 ± 256.6 *n* in the post-intervention group with the use of PVO. The *Power and Sample Size* programme was used with a confidence interval (CI) of 95%, an alpha level of 5%, and a test power of 90%. The sample calculation established a minimum of 6 participants.

### Procedures

**FIGURE 1:**
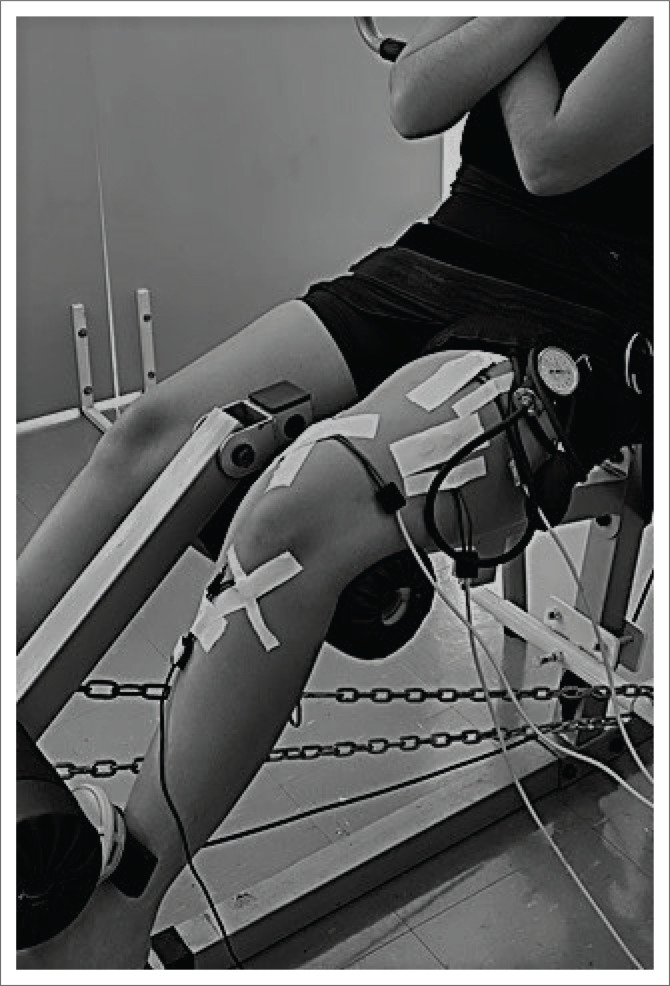
Maximum voluntary isometric contraction of the quadriceps to evaluate muscle strength and recruitment.

The participants were recruited and evaluated from June to December 2021. The evaluations were carried out at the Center for Research and Graduate Studies in Rehabilitation Sciences of the university. Initially, the participants received explanations about our study and after agreeing to participate, they signed the informed consent form and answered the sample characterisation questionnaire (Name, age, weight, height, body mass index [BMI], physical activity, modality and frequency). Subsequently, they were referred for evaluation with and without the previously randomised PVO. For randomisation, the numbers 1 or 2 were assigned for the conditions with and without PVO, and a random sequence was generated using www.random.org to determine the start condition for each participant (exercises with or without PVO). The PVO was performed using the Clinic Cuff–WCS of CardioMed^®^ (cuff size of 12.5 cm × 84 cm), located in the proximal region of the lower limb, with 200 mmHg of pressure. At each change of activity performed in the condition with PVO, the cuff was deflated for 5 min to perform revascularisation in the lower limb, as proposed by Centner et al. (2019). To evaluate muscle strength, the participants were seated with hips at 90° of flexion, a stabilisation band on the anterior thigh to control pelvic elevation, and the knee joint fixed at 60° of flexion to perform the maximum voluntary isometric contraction (MVIC) of the quadriceps, and upper limbs crossed over the thorax. To measure muscle strength, the participants were instructed to perform at the greatest possible knee extension force, with gradual onset and slow progression to maximum strength. The team of researchers provided verbal reinforcement to encourage greater muscle strength during the collection period. A portable dynamometer (MicroFET^®^ Hoggan Scientific, United States [US]) was positioned on the anterior and distal region of the lower limb (2 cm above the malleolar line) to measure quadriceps muscle strength in kilograms/force. The portable dynamometer was positioned against the structure of the extension chair (fixed by immovable iron chains), eliminating the evaluator’s resistance during the test (Figure 1). The MVIC had a duration of 6 s, and 3 repetitions were performed, with 1 min of rest between them. The mean muscle strength in kilograms in the threerepetitions was used for data analysis. Surface electromyography (EMG) was used to assess the quadriceps muscle recruitment with an 8-channel electromyograph (EMG model SAS1000V8/18_022884-R0A, EMG System®, Brazil). The EMG signal was captured with four pre-amplified active electrodes and filtered on a band-pass filter between 25 Hz and 450 Hz, with a sampling frequency of 2000 Hz.

To control and exclude interference in the electromyographic signals, all researchers, technicians, and those who needed to enter the collection environment were instructed not to use any electronic equipment, with cell phones turned off, and the computer and electromyograph were powered by a battery, without a connection to the power grid.

The skin was shaved and cleaned, and the surface electrodes were fixed on the rectus femoris, vastus lateralis, and vastus medialis oblique (VMO) muscles, according to the positioning rules proposed by *Surface Electromyography for the Non-Invasive Assessment of Muscle* (SENIAM), and the reference electrode was positioned on the anterior tuberosity of the homolateral tibia.

For data analysis, the root mean square (RMS) values were calculated for each muscle and normalised by the RMS peak to extract the percentage recruitment of each muscle. Finally, postural control was evaluated using a BIOMEC411 force platform (Serial number: NS_BIO1470, EMG System do Brasil®, SP Ltda.), which allows the quantitative analysis of body sway.

**FIGURE 2:**
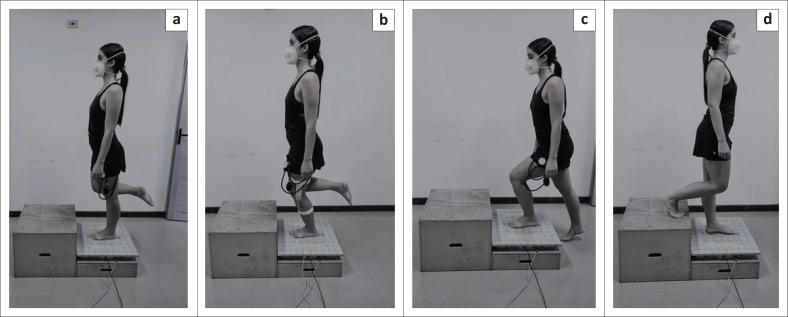
(a) Evaluation of postural control in a single-legged static position, (b) single-legged squat, (c) climbing and (d) descending stairs with partial vascular occlusion.

The signals were sampled at 100 Hz and filtered with a 35 Hz second-order Butterworth low-pass filter to cancel any interference. The activities were performed for the dominant lower limb (the one chosen to kick a ball) because the women were healthy, without complaints of pain. The activities evaluated were: (1) single-legged static position for 30 s (Figure 2a), (2) consecutive single-legged squats (Figure 2b) for 30 s, range of motion control of 0° – 40° of knee flexion, using an EMG System® Digital Goniometer, and (3) climbing (Figure 2c), and descending (Figure 2d) 2 steps, with the force platform positioned in the first step, to simulate the functional activity of ascending and descending stairs. All activities were performed 3 times, and the mean was considered for analysis.

The sequence of tests was established previously, always initiating with the evaluation of postural control (Figure 2) (to avoid the influence of fatigue generated in the maximal voluntary isometric contraction test [MVIC] on the evaluation of other outcomes) followed by muscle strength and recruitment (Figure 1) concomitantly.

### Statistical analysis

We applied the Shapiro-Wilk test to verify data normality. The Student’s t-test was used to compare the values in the conditions with and without vascular occlusion for the activities performed on the force platform, including static single-legged balance, single-legged squat, climbing and descending stairs, and the Wilcoxon test for the variables of strength and muscle recruitment. A CI of 95% was adopted, with a significance level of 5% (*p* < 0.05). We also investigated the correlation between quadriceps muscle strength and postural control variables for all functional activities, with and without PVO, using Spearman’s correlation test, considering correlations as insignificant (*r* < 0.30), low (*r* = 0.30–0.50), moderate (*r* = 0.50–0.70), high (*r* = 0.70–0.90), and very high (*r* > 0.90) (Mukaka 2012). All tests were analysed using Statistical Package For The Social Sciences (SPSS^®^) software (version 20, SPSS Inc., Chicago, IL).

### Ethical considerations

The Research Ethics Committee of the institution Human Research Ethics Committee of the State University of Londrina approved the research (number 80798017.7.0000.5231, Protocol number: 4.062.833). All participants signed an Informed Consent Form (ICF) and agreed to participate voluntarily in our study.

## Results

The participants (*n* = 14; women) evaluated in our study were characterised as healthy, as they did not present any reported diseases, pain, and/or dysfunctions. The participants presented a mean age of 23.15 (±1.81) years, weight of 60.15 (±11.3) kg, height of 1.65 (±0.06) m, and BMI of 22.04 (±3.58) kg/m^2^. The quadriceps muscle strength was similar when the participants were evaluated with and without PVO (Table 1). In addition, there was no difference in recruitment for the rectus femoris, VMO, and vastus lateralis muscles (Table 1).

**TABLE 1:** Results of quadriceps muscle strength and recruitment in women with and without partial vascular occlusion (*n* = 14).

Variables	Muscles	With PVO	Without PVO	*p*
Muscle strength (Kgf)	Quadriceps	35.18 (11.54)	36.50 (12.73)	0.45
Muscle recruitment (%RMS)	Rectus femoris	22.86 (18.79–29.29)	24.29 (20.01–29.83)	0.75
	VMO	23.54 (20.29–27.46)	23.72 (21.60–29.67)	0.27
	Vastus lateral	21.54 (19.26–25.17)	22.96 (19.54–27.53)	0.50

Note: The values are presented as medians and interquartile ranges, and the results were established by the Wilcoxon test.

PVO, partial vascular occlusion; KGF, kilogram-force; RMS, root mean square; VMO, vastus medialis oblique.

The results of postural control in the different tasks performed are described in Table 2.

**TABLE 2:** Results of postural control with and without partial vascular occlusion (*n* = 14).

Task	Postural control variables	With PVO		Without PVO		*p*
		Mean	s.d.	Mean	s.d.	
Static balance	AP Amplitude (cm)	4.43	1.14	4.19	0.95	0.49
	ML Amplitude (cm)	3.55	0.40	3.63	0.30	0.51
	AP Speed (cm/s)	3.07	0.64	5.17	0.84	0.001*
	ML Velocity (cm/s)	3.62	0.69	3.59	0.73	0.69
	COP Area (cm^2^)	9.95	3.06	10.03	2.94	0.92
Single-legged squat	AP Amplitude (cm)	7.19	1.78	6.60	1.01	0.11
	ML Amplitude (cm)	4.19	0.50	4.25	0.45	0.66
	AP Velocity (cm/s)	6.24	2.17	6.11	1.83	0.59
	ML Velocity (cm/s)	4.76	0.85	4.97	0.98	0.12
	COP Area (cm^2^)	21.86	7.45	19.92	4.96	0.15
Climbing stairs	AP Amplitude (cm)	16.79	3.54	18.41	3.61	0.21
	ML Amplitude (cm)	18.02	5.56	19.73	5.50	0.37
	AP Velocity (cm/s)	22.55	3.25	24.08	5.19	0.30
	ML Velocity (cm/s)	32.93	19.53	34.59	11.42	0.71
	COP Area (cm^2^)	232.40	75.81	245.32	76.15	0.63
Descending stairs	AP Amplitude (cm)	15.83	3.22	16.41	2.93	0.61
	ML Amplitude (cm)	19.30	5.28	18.59	5.06	0.75
	AP Velocity (cm/s)	32.65	6.45	33.14	5.99	0.82
	ML Velocity (cm/s)	37.65	10.93	37.52	10.45	0.97
	COP Area (cm^2^)	146.07	35.80	141.22	42.65	0.77

Note: The values are presented as means and standard deviations, compared by the Student’s *t*-test.

PVO, partial vascular occlusion; s.d., standard deviation; AP, anteroposterior; ML, medial-lateral, COP, centre of pressure.

* , difference between moments (*p*< 0.05).

The correlation between muscle strength and postural control variables with the use of PVO was negative and moderate for the ML amplitude (*r* = −0.54) and AP velocity (*r* = −0.59) in the task of climbing stairs, in other words, the higher the muscle strength, the lower the oscillation of postural control. Considering muscle strength without PVO, negative and moderate correlations were observed for AP amplitude (*r* = −0.60) and AP velocity (*r* = −0.55) in the single-legged squatting task and in the descending stairs for ML velocity (*r* = −0.69). A strong correlation was found only for AP velocity (*r* = −0.76) in the descending stairs without PVO.

## Discussion

Our study is the first to analyse the influence of PVO on strength, quadriceps muscle recruitment, and postural control in healthy women. The results contribute to clarifying and affirming that the use of PVO in static exercises, mini squats, and climbing and descending stairs does not alter the strength and muscle activation of the quadriceps and postural control in healthy women, and that PVO does not cause balance deficits during use, highlighting the safety of using PVO in functional activities, during training, and for prevention and rehabilitation.

The use of PVO devices has become increasingly frequent in physical activity practice sites such as gyms, training centres, and rehabilitation centres, which emphasises the importance of studies that ensure the criteria for its use and safety. The practice of exercises with PVO decreases the time to exhaustion, explained through the processes of muscle fatigue that occur early because of the lack of oxygen and failure in oxidative capacity, which causes an accelerated decline in muscle fibre strength (Willberg et al. 2021). Therefore, there is a need to identify possible deficits, for example, the quality of movement during performance with the PVO device, to ensure that its use does not lead to changes during exercise.

Several studies have shown favourable results for muscle strength gain through PVO in different populations (Ferlito et al. 2020; Wortman et al., 2021). May et al. (2022) found that muscle endurance training with and without PVO increased the muscle strength of knee extension and flexion and muscle cross-sectional area. Copithorne and Rice (2019) established that applying PVO in the elbow flexor muscles induces the shortest time to task failure, characterised as induced peripheral fatigue, however, two minutes after the release of blood flow. An alteration in the preferential metabolic energy production of type I oxidative fibres caused by the ischaemic environment and the rapid recovery (0 min - 2 min) may justify the early failure. The fast recovery may also be associated with reperfusion and a hyperaemic response after restriction, with renewed oxygen supply for the metabolism of type I fibres (Copithorne & Rice 2019).

The results of Copithorne and Rice (2019) reinforce the importance and concern of understanding the neuromuscular processes involved during the performance of exercises with PVO when the muscles are active in a hypoxic environment. Contributing to the findings on exercises performed with PVO, our study evaluated the muscle strength of the quadriceps and recruitment of the vastus medialis, lateralis, and rectus femoris muscles during MVIC, with and without PVO, as occurs in training for strength gain in gyms and rehabilitation processes. Our results showed no difference in muscle strength or recruitment with or without PVO, which can be considered favourable and may mean that even in the anaerobic environment caused by PVO, the muscle maintained the same muscle strength and recruitment without impairing the exercise.

Studies have shown that postural control is crucial for performing different activities of daily living (Duarte & Freitas 2010). In addition, evaluating postural control can highlight balance deficits related to proprioception and postural adjustments of the neuromuscular system (Da Silva et al. 2013). Our results showed that PVO exercises did not impair postural control and improved AP velocity in the single-legged static position with the need for a shorter time to adapt the centre of pressure in the anteroposterior (AP) direction. Therefore, the exercises performed with PVO are not harmful to postural control. Corroborating our results, Willberg et al. (2021) evaluated the influence of PVO on the static and dynamic postural control of physically active individuals and asserted that although the condition with PVO leads to greater deoxygenation and less time required for exhaustion, postural control, and the ability to regain stability after the disturbance was not affected. However, that study evaluated postural control in a bipedal position with knee flexion at 110° and not during the functional activity of climbing and descending stairs, which are movements of daily life, and when altered could cause compensations and biases in movements and postures. It is important to highlight that it is extremely important to evaluate possible changes in postural control during functional activities, such as going up and down steps, which are performed frequently and are more related to the reality of human movement.

Finally, our results showed a moderate and negative correlation between quadriceps muscle strength and postural control variables when the participants used PVO. In addition, moderate and strong correlations were observed when strength and postural control were evaluated without PVO. These results show that the increase in muscle strength decreases postural control oscillations, agreeing with the studies by Carcelén-Fraile et al. (2021) and Forte et al. (2021), and highlighting that exercise to increase muscle strength, with or without PVO, can improve postural control.

Our study presents limitations such as the time of analysis of the quadriceps muscle contraction of 6 s, which did not cause muscle exhaustion, and further studies could analyse a longer time of muscle contraction or even muscle fatigue in the task performed. In addition, the sample comprised women without complaints and dysfunctions, and the results can be extrapolated only for muscle training or prevention activities and not for rehabilitation. We also considered the sample to be small, which may be related to type II errors. Notably, the results found are important for clinical practice because, during the use of the PVO, there are no deficits in postural control and strength and muscle recruitment of the quadriceps, with a lower risk of injuries. Future studies should be conducted that develop activities more realistic to the practice of resistance training, for example, the time of vascular occlusion during training, to reinforce these results. Two distinct groups could also be investigated (with and without PVO) and between men and women.

## Conclusion

Partial vascular occlusion of the proximal region of the thigh did not alter the strength and muscle recruitment of the quadriceps, or the postural control of young, healthy women performing functional activities in a static posture, squatting, and climbing and descending stairs.

## References

[CIT0001] Álvarez, C.B., Santamaría, P.I.K., Fernández-Matías, R., Pecos-Martín, D., Achalandabaso-Ochoa, A., Fernández-Carnero, S. et al., 2021, ‘Comparison of blood flow restriction training versus non-occlusive training in patients with anterior cruciate ligament reconstruction or knee osteoarthritis: A systematic review’, *Journal of Clinical Medicine* 10(1), 1–23. 10.3390/jcm10010068 PMC7796201 33375515

[CIT0002] Carcelén-Fraile, M.d.C., Aibar-Almazán, A., Martínez-Amat, A., Brandão-Loureiro, V., Jiménez-García, J.D., Castellote-Caballero, Y. et al., 2021, ‘Qigong for muscle strength and static postural control in middle-aged and older postmenopausal women: A randomized controlled trial’, *Frontiers in Medicine* 8, 1–8. 10.3389/fmed.2021.784320 PMC8692287 34957157

[CIT0003] Centner, C., Wiegel, P., Gollhofer, A. & König, D., 2019, ‘Effects of blood flow restriction training on muscular strength and hypertrophy in older individuals: A systematic review and meta-analysis’, *Sports Medicine* 49(1), 95–108. 10.1007/s40279-018-0994-1 30306467 PMC6349784

[CIT0004] Clark, B.C., Manini, T.M., Hoffman, R.L., Williams, P.S., Guiler, M.K., Knutson, M.J. et al., 2011, ‘Relative safety of 4 weeks of blood flow-restricted resistance exercise in young, healthy adults’, *Scandinavian Journal of Medicine and Science in Sports* 21(5), 653–662. 10.1111/j.1600-0838.2010.01100.x 21917016 PMC6152804

[CIT0005] Cognetti, D.J., Sheean, A.J. & Owens, J.G., 2022, ‘Blood flow restriction therapy and its use for rehabilitation and return to sport: Physiology, application, and guidelines for implementation’, *Arthroscopy, Sports Medicine, and Rehabilitation* 4(1), e71–e76. 10.1016/j.asmr.2021.09.025 35141538 PMC8811521

[CIT0006] Copithorne, D.B. & Rice, C.L., 2019, ‘The effect of blood flow occlusion during acute low-intensity isometric elbow flexion exercise’, *European Journal of Applied Physiology* 119(3), 587–595. 10.1007/s00421-019-04088-8 30734842

[CIT0007] Da Silva, R.A., Bilodeau, M., Parreira, R.B., Teixeira, D.C. & Amorim, C.F., 2013, ‘Age-related differences in time-limit performance and force platform-based balance measures during one-leg stance’, *Journal of Electromyography and Kinesiology* 23(3), 634–639. 10.1016/j.jelekin.2013.01.008 23403137

[CIT0008] Duarte, M. & Freitas, S.M.S., 2010, ‘Revisão sobre posturografia baseada em plataforma de força para avaliação do equilíbrio’, *Revista Brasileira de Fisioterapia* 14(3), 183–192. 10.1590/S1413-35552010000300003 20730361

[CIT0009] Early, K.S., Rockhill, M., Bryan, A., Tyo, B., Buuck, D. & McGinty, J., 2020, ‘Effect of blood flow restriction training on muscular performance, pain and vascular function’, *International Journal of Sports Physical Therapy* 15(6), 892–900. 10.26603/ijspt20200892 33344005 PMC7727422

[CIT0010] Ellefsen, S., Hammarström, D., Strand, T.A., Zacharoff, E., Whist, J.E., Rauk, I. et al., 2015, ‘Blood flow-restricted strength training displays high functional and biological efficacy in women: A within-subject comparison with high-load strength training’, *American Journal of Physiology-Regulatory, Integrative and Comparative Physiology* 309(7), 767–779. 10.1152/ajpregu.00497.2014 PMC4666930 26202071

[CIT0011] Ferlito, J.V., Pecce, S.A.P., Oselame, L. & De Marchi, T., 2020, ‘The blood flow restriction training effect in knee osteoarthritis people: A systematic review and meta-analysis’, *Clinical Rehabilitation* 34(11), 1378–1390. 10.1177/0269215520943650 32772865

[CIT0012] Forte, R., Ditroilo, M., Boreham, C. & De Vito, G., 2021, ‘Strength training and gross-motor skill exercise as interventions to improve postural control, dynamic functional balance and strength in older individuals’, *Journal of Sports Medicine and Physical Fitness* 61(12), 1570–1577. 10.23736/S0022-4707.21.11947-4 33480515

[CIT0013] Hughes, D.C., Ellefsen, S. & Baar, K., 2018, ‘Adaptations to endurance and strength training’, *Cold Spring Harbor Perspectives in Medicine* 8(6), a029769. 10.1101/cshperspect.a029769 28490537 PMC5983157

[CIT0014] Hughes, X.L., Stephen, X. & Patterson, D., 2020, ‘The effect of blood flow restriction exercise on exercise-induced hypoalgesia and endogenous opioid and endocannabinoid mechanisms of pain modulation’, *Journal of Applied Physiology* 128(4), 914–924. 10.1152/japplphysiol.00768.2019 32105522

[CIT0015] Humes, C., Aguero, S., Chahla, J. & Foad, A., 2020, ‘Blood flow restriction and its function in postoperative anterior cruciate ligament reconstruction therapy: Expert opinion’, *Archives of Bone and Joint Surgery* 8(5), 570–574. 10.22038/abjs.2020.42068.2145 33088857 PMC7547166

[CIT0016] Korakakis, V., Whiteley, R. & Epameinontidis, K., 2018, ‘Blood flow restriction induces hypoalgesia in recreationally active adult male anterior knee pain patients allowing therapeutic exercise loading’, *Physical Therapy in Sport* 32, 235–243. 10.1016/j.ptsp.2018.05.021 29879638

[CIT0017] Lixandrão, M.E., Ugrinowitsch, C., Berton, R., Vechin, F.C., Conceição, M.S., Damas, F. et al., 2018, ‘Magnitude of muscle strength and mass adaptations between high-load resistance training versus low-load resistance training associated with blood-flow restriction: A systematic review and meta-analysis’, *Sports Medicine* 48(2), 361–378. 10.1007/s40279-017-0795-y 29043659

[CIT0018] May, A.K., Russell, A.P., Della Gatta, P.A. & Warmington, S.A., 2022, ‘Muscle adaptations to heavy-load and blood flow restriction resistance training methods’, *Frontiers in Physiology* 13, 837697. 10.3389/fphys.2022.837697 35185627 PMC8850930

[CIT0019] Miller, B.C., Tirko, A.W., Shipe, J.M., Sumeriski, O.R. & Moran, K., 2021, ‘The systemic effects of blood flow restriction training: A systematic review’, *International Journal of Sports Physical Therapy* 16(4), 978–990. 10.26603/001c.25791 34386277 PMC8329318

[CIT0020] Mukaka, M.M., 2012, ‘Statistics corner: A guide to appropriate use of correlation coefficient in medical research’, *Malawi Medical Journal* 24(3), 69–71. 23638278 PMC3576830

[CIT0021] Nakajima, T., Takano, H., Kurano, H., Iida, N., Kubota, T., Yasuda, M. et al., 2007, ‘Effects of KAATSU training on haemostasis in healthy subjects’, *International Journal of KAATSU Training Research* 3, 11–20. 10.3806/ijktr.3.11

[CIT0022] Ratamess, N.A., Alvar, B.A., Evetoch, T.K., Housh, T.J., Kibler, B., Kraemer, W.J. et al., 2009, ‘Progression models in resistance training for healthy adults’, *Medicine and Science in Sports and Exercise* 41(3), 687–708. 10.1249/MSS.0b013e3181915670 19204579

[CIT0023] Slysz, J., Stultz, J. & Burr, J.F., 2016, ‘The efficacy of blood flow restricted exercise: A systematic review & meta-analysis’, *Journal of Science and Medicine in Sport* 19(8), 669–675. 10.1016/j.jsams.2015.09.005 26463594

[CIT0024] Takarada, Y., Takazawa, H., Sato, Y., Takebayashi, S., Tanaka, Y. & Ishii, N., 2000, ‘Effects of resistance exercise combined with moderate vascular occlusion on muscular function in humans’, *Journal of Applied Physiology* 88(6), 2097–2106. 10.1152/jappl.2000.88.6.2097 10846023

[CIT0025] Willberg, C., Zentgraf, K. & Behringer, M., 2021, ‘The effect of lower-body blood flow restriction on static and perturbated stable stand in young, healthy adults’, *Frontiers in Human Neuroscience* 15, 756230. 10.3389/fnhum.2021.756230 34744667 PMC8570169

[CIT0026] Wortman, R.J., Brown, S.M., Savage-Elliott, I., Finley, Z.J. & Mulcahey, M.K., 2021, ‘Blood flow restriction training for athletes: A systematic review’, *American Journal of Sports Medicine* 49(7), 1938–1944. 10.1177/0363546520964454 33196300

